# Editorial: Racism as a Public Health Crisis: From Declaration to Action

**DOI:** 10.3389/fpubh.2022.893804

**Published:** 2022-05-31

**Authors:** Georges C. Benjamin, Camara Phyllis Jones, Regina Davis Moss

**Affiliations:** ^1^Satcher Health Leadership Institute and Cardiovascular Research Institute, American Public Health Association, Washington, DC, United States; ^2^Community Health & Preventive Medicine, Morehouse School of Medicine, Atlanta, GA, United States

**Keywords:** racism, public health, emergency, policy, community-based participatory research (CBPR), research, data

The movement for racial and social justice that swept the United States in recent years has drawn greater attention to life-threatening inequities. The issue of how race-related health inequities are affecting disadvantaged groups has received sharper focus, bringing widespread promises of reform along with it.

Nearly 250 declarations of racism as a public health crisis have passed in states, cities, town councils, county and education boards, and public health entities. While resolutions and formal statements themselves are not necessarily legally enforceable, they are an important first step in shifting a narrative that can drive meaningful changes to public health programming, policies, and resource allocation.

Racism is a system of structuring opportunity and assigning value based on the social interpretation of how one looks (which is what we call “race”), which unfairly disadvantages some individuals and communities, unfairly advantages others, and saps the strength of the whole society through the waste of human resources. Improving health and addressing inequities requires acknowledging that racism exists, correcting misconceptions that fuel racism, and facilitating healing in communities.

In this Research Topic feature, we share what many experts have to say about racialized impacts on health as well as innovations that can foster and sustain transformational change in policies and practices that are driving the social determinants of health and wellbeing. Several underlying themes crisscross the many articles and Editorials including, the need to improve the quality of health data; transforming how we communicate about our past, present, and future; practices and policies that sustain false beliefs in hierarchy; progress of national and state-level legislation to address health inequities, and the importance of community-based participatory research approaches for authentic voices. Many of the manuscripts share real-life examples of how racism and health equity are being addressed across the U.S.

County and city leaders who are looking for practical ways to advance health and racial equity now have a new resource that can serve as a roadmap. In 2021, the American Public Health Association, the de Beaumont Foundation, and the National Collaborative for Health Equity launched *Healing Through Policy: Creating Pathways to Racial Justice*, an initiative that offers local leaders a suite of policies and practices that are being implemented across the country to help advance health, racial equity, and justice. *Healing Through Policy* uses the Truth, Racial Healing and Transformation (TRHT) framework, a comprehensive, national, and community-based process to plan for and bring about change, and to address the historic and contemporary effects of racism. First introduced by the W. K. Kellogg Foundation in 2017, the TRHT framework is used by communities around the nation and on dozens of college campuses to promote racial healing and relationship building. The suite is available at: www.debeaumont.org/healing-through-policy.

## Author Contributions

All authors listed have made a substantial, direct, and intellectual contribution to the work and approved it for publication.

## Conflict of Interest

GB and RD were employed by American Public Health Association, Washington, DC, United States. The remaining author declares that the research was conducted in the absence of any commercial or financial relationships that could be construed as a potential conflict of interest.

## Publisher's Note

All claims expressed in this article are solely those of the authors and do not necessarily represent those of their affiliated organizations, or those of the publisher, the editors and the reviewers. Any product that may be evaluated in this article, or claim that may be made by its manufacturer, is not guaranteed or endorsed by the publisher.

